# Editorial: Women in gynecological oncology 2021

**DOI:** 10.3389/fonc.2023.1220467

**Published:** 2023-05-24

**Authors:** Mayur Virarkar, Priya Ranjit Bhosale

**Affiliations:** ^1^ Department of Radiology, University of Florida, Jacksonville, FL, United States; ^2^ Department of Diagnostic Radiology, University of Texas MD Anderson Cancer Center, Houston, TX, United States

**Keywords:** gynecological, oncology, original research, treatment, review

## Introductions

This editorial summarizes the contributions to the Frontiers Research Topic “*Women in gynecological oncology 2021*”. The cohort comprises 33 articles with one clinical trial, 25 original research, 6 systemic analyses, and 1 review article. They were published from September 2021 through August 2022 period. As of May 2023, the article’s impact analysis has over 61,000 views, with US and China leading viewers. The editorial has over 300 authors, and the article view ranges from 1000- 4000. These Research Topic aimed to explore recent developments in the area of gynecological oncology diagnosis, treatment, and the latest research.

## Clinical trial

The only clinical trial (ChiCTR2000035061) in the editorial by Wang et al. comprised a screening program to investigate the prevalence of human papillomavirus (HPV) and HPV genotype distribution to reveal cervical cancer and its precursor, which led to morbidity among women in northern Tibet. They reported total HPV infection rate among women in Nagqu was 13.42%. In addition, they identified the five most common HPV genotypes, accounting for more than 60% of all HPV infections in Nagqu people, were HPV 16, 58, 31, 18, and 52.

## Original articles

While summarising all the original research articles in the cohort will be beyond the scope of the editorial. We have discussed the four most viewed articles below.

Wei et al. analyzed the heterogeneity from transcriptional to immune infiltration between HPV+ and HPV- samples by Single-cell RNA sequencing (scRNA-seq) and bulk RNA sequencing. The study revealed heterogeneity from the transcriptional state to immune infiltration. They believe that single-cell transcriptomics is a powerful tool to explore the heterogeneity of HPV+ and HPV- tumors, which can facilitate assessing the impact of HPV infection on cancer biology by identifying gene markers for diagnosis, prognosis, and therapy.

The study by Ni et al. aimed to perform homologous recombination deficiency (HRD) testing of ovarian cancer in the real world in China and correlate HRD status and clinical characteristics with therapeutic outcomes. The results demonstrated that patients with high HRD scores tended to enrich in BRCA mutation and HRR mutation. The study is the first real-world study of Next-Generation Sequencing (NGS) -based HRD in Chinese ovarian and fallopian tube cancer patients.

The pilot study by George et al. evaluates population-level patterns of ovarian cancer in Black women globally and locally and describes the correlative patterns between age and histologic distribution in our Transatlantic Gynecologic Cancer Research Consortium. -member cohorts. The analysis revealed that the burden of ovarian cancer is increasing annually. By 2040, there is an expected 49.6-86.8% increase in ovarian cancer incidence in Africa, Latin America, and the Caribbean, which is much greater than the modest 9.6-25.9% increase expected among the predominantly White regions of Europe and North America.

`Xing et al. aimed to screen ferroptosis- and immune-related differentially expressed genes (FI-DEGs) to identify appropriate prognostic signatures for cervical squamous cell carcinoma patients endocervical adenocarcinoma (CESC). They found that Dual oxidase 1 (DUOX1) expression increased in CESC patients and suggested that DUOX1 may play different roles in different cancers, and the abnormal DUOX1 expression was only the result of CESC.

## Review article

The only review article by Zannoni et al. focused on summarizing the histological features and immunohistochemical and molecular biomarkers useful to characterize endometrial cancer. The authors believe molecular classifiers should be combined with clinical risk groups and pathological parameters in an integrated histo-molecular approach to better discriminate outcomes for different patients.

## Systemic review

The systemic analysis primarily focused on treatment, with one focusing on the association between endometriosis and ovarian cancer (Chen et al.).

Zhang et al. performed a meta-analysis to compare and evaluate medium- (3-year) and long-term (5-year) survival outcomes in patients with early-stage cervical cancer who underwent MIS (laparoscopic or robot-assisted radical hysterectomy) or Abdominal radical hysterectomy (ARH). The meta-analysis included 48 studies with 23346 patients and reported that early-stage cervical cancer undergoing MIS had a poorer prognosis than those undergoing ARH.

Inzani et al. reviewed the post-treatment pathological scoring systems for gynecological malignancies. According to current guidelines, the chemotherapy response score (CRS) system applied to the omental tissue is mandatory in pathological reports to evaluate the response of post-neoadjuvant high-grade serous carcinoma. Pressurized intraperitoneal aerosol chemotherapy (PIPAC) has emerged in recent years as a novel method of intraperitoneal drug administration to treat inoperable peritoneal metastasis from different origins. A 4-degree system called peritoneal regression grading score (PRGS) is used to monitor histological response on biopsies made during PIPAC procedures.

Maiorano et al. systematically reviewed the clinical trials regarding immune checkpoint inhibitors (ICIs) for the treatment of advanced Endometrium Cancer (EC). They reported that ICIs were an effective option for pretreated advanced EC patients with tolerable profiles and combining ICIs and Tyrosine kinase inhibitors (TKIs) are more effective in Microsatellite stable (MSS) women than monotherapy and pretreated advanced/recurrent EC.

Wang et al. meta-analysis evaluated the efficacy and safety of a placebo during the maintenance therapy of ovarian cancer (OC) patients in randomized controlled trials (RCTs). In total, 41 articles with 20,099 patients were included in this meta-analysis and reported that placebo did not improve or reduce the progression-free survival (PFS) and overall survival (OS) benefits of OC patients in RCTs but increased the incidences of common adverse effects (AEs).

Ren et al. performed a network pharmacology analysis to explore traditional Chinese medicine’s potential pharmacological mechanisms and targets (TCM) in treating gynecological cancer (GC). The study, which included 11 RCTs of 863 GC patients, showed that TCM plus chemotherapy could provide more durable disease control and improve GC patients’ Quality of life (QOL) without substantially increasing AEs compared with chemotherapy alone.

In the meta-analysis conducted by Chen et al. to uncover the differences in PFS and OS within endometriosis-associated ovarian cancer (EAOC) and non-EAOC patients. Twenty-one studies involving 38641 patients were included and reported that EAOC patients tended to have better OS and PFS than non-EAOC patients.

## Conclusion

This Research Topic is very insightful and helpful in understanding the accurate research methodology and getting up to date with biomarkers in gynecological oncology. The authors hope to impact the reader’s interest in respective Research Topic and will push them to pursue their future endeavors in gynecological oncology.

## Author contributions

PB and MV contributed to the conception and design of the study. PB and MV wrote sections of the manuscript. All authors contributed to the manuscript revision, and read, and approved the submitted version.

